# Effect of inspiratory muscle training with load compared with sham training on blood pressure in individuals with hypertension: study protocol of a double-blind randomized clinical trial

**DOI:** 10.1186/s13063-016-1514-y

**Published:** 2016-08-02

**Authors:** Simone Regina Posser, Carine Cristina Callegaro, Marina Beltrami-Moreira, Leila Beltrami Moreira

**Affiliations:** 1 Graduate Studies Program in Health Sciences: Cardiology and Cardiovascular Sciences, Universidade Federal do Rio Grande do Sul (UFRGS), Av. Jerônimo de Ornelas, 721, Porto Alegre, RS 90040-341 Brazil; 2 Graduate Program in Integral Attention to Health (PPGAIS- UNICRUZ/UNIJUI). Universidade de Cruz Alta, Rodovia Municipal Jacob Della Méa, Km 5.6, Cruz Alta, RS 98020-290 Brazil; 3 Hospital de Clínicas de Porto Alegre, Rua Ramiro Barcelos, 2350, room 943, 90035-903 Porto Alegre, RS Brazil

**Keywords:** Blood pressure, Clinical trial, Fitness, Hypertension, Physical therapy, Respiratory exercises, Sympathetic nervous system

## Abstract

**Background:**

Hypertension is a complex chronic condition characterized by elevated arterial blood pressure. Management of hypertension includes non-pharmacologic strategies, which may include techniques that effectively reduce autonomic sympathetic activity. Respiratory exercises improve autonomic control over cardiovascular system and attenuate muscle metaboreflex. Because of these effects, respiratory exercises may be useful to lower blood pressure in subjects with hypertension.

**Methods/design:**

This randomized, double-blind clinical trial will test the efficacy of inspiratory muscle training in reducing blood pressure in adults with essential hypertension. Subjects are randomly allocated to intervention or control groups. Intervention consists of inspiratory muscle training loaded with 40 % of maximum inspiratory pressure, readjusted weekly. Control sham intervention consists of unloaded exercises. Systolic and diastolic blood pressures are co-primary endpoint measures assessed with 24 h ambulatory blood pressure monitoring. Secondary outcome measures include cardiovascular autonomic control, inspiratory muscle metaboreflex, cardiopulmonary capacity, and inspiratory muscle strength and endurance.

**Discussion:**

Previously published work suggests that inspiratory muscle training reduces blood pressure in persons with hypertension, but the effectiveness of this intervention is yet to be established. We propose an adequately sized randomized clinical trial to test this hypothesis rigorously. If an effect is found, this study will allow for the investigation of putative mechanisms to mediate this effect, including autonomic cardiovascular control and metaboreflex.

**Trial registration:**

ClinicalTrials.gov NCT02275377. Registered on 30 September 2014.

**Electronic supplementary material:**

The online version of this article (doi:10.1186/s13063-016-1514-y) contains supplementary material, which is available to authorized users.

## Background

Essential hypertension is a multifactorial disease characterized by chronically elevated systolic blood pressure (≥140 mmHg) or diastolic blood pressure (≥90 mmHg) [1–4]. Hypertension is a major risk factor for target-organ dysfunction, cardiovascular disease, and premature mortality [3, 4]. The prevalence of hypertension in Brazil has been decreasing over the past three decades, but 30 % of persons over 18 years of age still bear this condition [5].

The autonomic nervous system is a major determinant of systemic blood pressure and it might be a therapeutic target in hypertension [6, 7]. Hyperactivity of the sympathetic nervous system plays a role in abnormal elevations of blood pressure [6–8]. Blood pressure variability is increased in hypertensive persons. This phenomenon has been linked to desensitization of baroreceptors, leading to an anomalous autonomic response [9, 10]. Beta-adrenergic receptor blockers are an example of an effective pharmacologic approach to target the autonomic nervous system in hypertension [11].

Respiratory exercises are a non-pharmacologic intervention that might modulate autonomic nervous system activity and reduce blood pressure [12, 13]. An exercise composed of controlled respiratory patterns with a slow respiratory rate has improved autonomic control and reduced blood pressure in hypertensive subjects [12]. Inspiratory muscle training – a different modality of respiratory exercise – was tested in one randomized clinical trial that included 13 participants [13]; in this study, inspiratory muscle training reduced 24 h systolic blood and diastolic blood pressure by 7.9 and 5.5 mmHg, respectively, and also improved autonomic cardiovascular control. Respiratory muscle training might also have reduced sympathetic activity through attenuation of muscle metaboreflex [14, 15].

## Methods/design

### Study aim

This study aims to test the efficacy of inspiratory muscle training in reducing blood pressure in subjects with essential hypertension. It will further investigate the effects of this intervention on inspiratory metaboreflex – a putative mechanism for blood pressure reduction. The conceptual hypothesis is that inspiratory muscle training reduces mean 24 h blood pressure through modulation of sympathetic nervous system activity and muscle metaboreflex.

### Study design

**Fig. 1:**
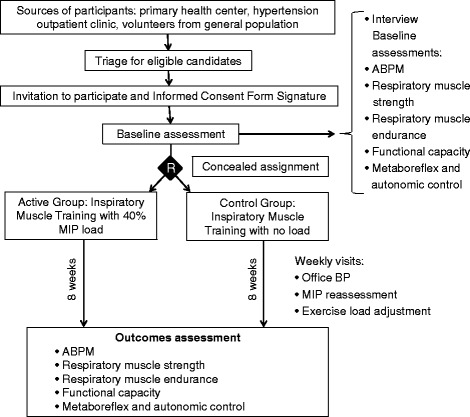
Study flow diagram. *BP* blood pressure, *ABPM* ambulatory blood pressure monitoring, *MIP* maximum inspiratory pressure; *R* randomization

This is a double-blind, parallel-group, sham-controlled randomized clinical trial. Participants are randomly assigned to receive inspiratory muscle training with a load equivalent to 40 % of maximum inspiratory pressure or inspiratory muscle training sham (no load) for 8 weeks. The study flowchart is depicted in Fig. 1. Participants are followed on weekly visits. Study outcomes are assessed at the end of the eighth week. The study timeline is summarized in Additional file 1: Figure S1, where detailed information on methods of assessment and protocols can be found.

### Study context

Participants will be recruited from patients receiving follow-up care at the hypertension clinic or the primary health care center at the Hospital de Clínicas de Porto Alegre, a tertiary, university-affiliated teaching hospital. Public announcements (radio, newspapers, and website) will also call for volunteers among residents of the city of Porto Alegre.

### Eligibility criteria

The study includes both male and female subjects, aged ≥35 and <65 years, previously diagnosed with essential hypertension, with a systolic blood pressure ≥140 mmHg or diastolic blood pressure ≥90 mmHg. Subjects receiving no pharmacologic therapy or taking only a thiazide diuretic are eligible to enter the study. Participants must have body mass index <30 kg/m^2^ [16], practice <150 min of moderate or intense physical activity per week, according to the International Physical Activity Questionnaire [17, 18], and have inspiratory muscle strength ≥70 % of predicted [19].

Participants are excluded from the study if they have: office blood pressure ≥160/100 mmHg; severe dyspnea; diabetes mellitus; orthopedic, musculoskeletal, or neurologic limitations; significant cognitive impairment; a current or past history of deep venous thrombosis; a history of myocardial infarction or stroke in the previous 6 months; congestive heart failure; unstable angina; or pulmonary disease of any etiology (including asthma and chronic obstructive pulmonary disease); or if they are active smokers. Women who are pregnant or breastfeeding are excluded from this study. Subjects are not eligible if they participated in another clinical trial in the previous 30 days, or if there is any other condition that might impair their ability to participate in the study or follow the protocol, at the discretion of the recruiter.

Participants are also directed not to change pharmacologic or non-pharmacologic treatment for hypertension (including changes in prescription or changes in the of level physical activity) during their participation in this study.

### Study outcomes

The co-primary outcomes measures are changes from baseline to end of study in mean 24 h systolic and diastolic blood pressure, as evaluated by ambulatory blood pressure monitoring.

Secondary outcome measures are changes from baseline to end of study in mean daytime systolic and diastolic blood pressure; changes from baseline to end of study in mean nighttime systolic and diastolic blood pressure; heart rate variability; systolic blood pressure variability; aerobic capacity; inspiratory metaboreflex; and autonomic control.

### Randomization, allocation, blinding, and confidentiality

A randomization sequence has been generated using Randomization.org in blocks of four to either group 1 – inspiratory muscle training with 40 % of maximal inspiratory pressure – or group 2 – sham inspiratory muscle training. An independent person not involved in this study possesses the computer-generated randomization sequence. A research assistant not involved in participant assessment obtains the code and sets the load in each device, according to randomization. A black tape hides the loading spring from the participant and from the investigators assessing outcomes.

There are no foreseen circumstances when un-blinding is permissible. When data collection is completed, the person responsible for the randomization list will provide assignment information to the investigators. Patient’s forms and other data sources are kept in a locked cabinet. Data are anonymized when entered into the database to protect confidentiality. Only investigators formally associated with the project will have access to the final study database.

### Studied variables and methods of assessment

Participants are assessed as described in Additional file 1. Briefly, baseline physical activity is quantified using the International Physical Activity Questionnaire long form [20]. Body mass index is calculated as mass (kg) divided by the square of the height (m) and categorized as recommended by the World Health Organization [21]. Ambulatory blood pressure monitoring is performed with Spacelabs 90207 devices (Redmond, WA, USA). The protocol includes blood pressure measurement every 15 min during the daytime (6 a.m. to 10 p.m.) and every 20 min during the nighttime (10 p.m. to 6 a.m.) [22]. Functional capacity is assessed during exercise through O_2_ and CO_2_ expiration fraction in a metabolic system. Maximum oxygen consumption in each breath is recorded in ml/(Kg/min) [23, 24].

Maximum inspiratory and maximum expiratory pressure serve as indicators of inspiratory and expiratory muscle strength, respectively. Maximum inspiratory and maximum expiratory pressure are assessed using a digital pressure manometer (MVD-300, Microhard System, Globalmed, Porto Alegre, Brazil) [25, 26].

Inspiratory muscle endurance is determined using the incremental test proposed by Martyn et al. [27] using the Powerbreathe Plus® system (London, UK). The test is stopped when the subject cannot open the inspiratory valve or desires to stop the test owing to respiratory exhaustion (quantified using the modified Borg scale).

Blood pressure variability, heart rate variability, and the cardiac vagal tone are assessed to evaluate autonomic cardiovascular control. Non-invasive continuous blood pressure curves, heart rate, and electrocardiogram tracing are obtained simultaneously at a frequency of 1000 Hz using a Biopac MP150 (Biopac, California, EUA). Data are registered in a computer with biologic signal conversion capabilities.

The muscle metaboreflex is the adaptive blood flow redistribution from the peripheral circulation into the vascular bed of metabolically active (exercised) muscles [28]. The metaboreflex intensity is inversely related to one’s fitness. Inspiratory muscle metaboreflex is assessed by causing diaphragmatic fatigue and detecting peripheral blood flow reduction [28, 29]. Monitored parameters include: oxygen saturation, carbon dioxide partial expiratory tension, mean arterial pressure, and venous occlusion plethysmography. A detailed protocol can be found in Additional file 1.

### Intervention

Participants are extensively instructed on how to perform home-based inspiratory muscle training assisted by a Power Breathe Plus® (London, UK) device. For participants’ safety, research staff are responsible for device hygiene before and after the intervention period. To perform the exercise training, subjects should be seated, obstruct the nose with a nasal clip, and breathe in and out through the mouthpiece. The goal is to keep diaphragmatic breathing at 10–15 respirations per minute for a total of 30 min per day, 7 days a week.

Participants receiving active inspiratory muscle training (intervention group) perform the exercises with a load equivalent to 40 % of maximal inspiratory pressure. Those receiving the sham intervention (the control group) follow the same instructions but have no load applied to the respiratory exercises. The sham intervention was chosen as comparator because it is the most similar intervention available. It allows for blinding of investigators and participants, and is not expected to reproduce the effects of loaded exercises.

The complete study protocol consists of 56 daily training sessions completed over 8 weeks. During the scheduled weekly visit to the study center, participants are questioned about possible discomforts associated with the intervention, which may include: headaches, nausea, intense muscle fatigue, muscle cramps, and chest pain. Respiratory muscle strength is then reassessed; inspiratory threshold loading is adjusted accordingly, keeping a 40 % maximum inspiratory load for the intervention group throughout the study. The staff member who receives information about treatment allocation and adjusts the load neither participates in any assessments nor communicates with participants.

Participants in both groups receive a journal to register date, time, duration of training session, and possible symptoms or discomforts associated to the intervention. This serves as a mean to stimulate and estimate adherence to intervention. Adherence to the protocol is also reinforced on weekly follow-up phone calls.

### Sample size calculation

Sample size was estimated based on a small randomized clinical trial on the effect of inspiratory muscle training on blood pressure of hypertensive subjects in Brazil [13]. That study found an 8 mmHg difference in systolic blood pressure between the active treatment and the control group (standard deviation, 10 mmHg). To detect this difference with power of 80 %, and assuming an alpha error of 5 %, the study should include 26 participants per group. We opted for a 20 % inflation of this number to account for loss of follow-up and consent withdrawal – therefore, a total of 60 participants will be included and assigned to either intervention or sham treatment.

### Data management and statistical analysis plan

Data recorded in printed forms are kept in locked cabinets. Information is entered on Microsoft Excel spreadsheets by one person and reviewed by another. Numeric continuous variables, including blood pressure in the primary outcome measures, will be described as mean ± standard deviation or as median (interquartile range). Comparison between groups will be performed using Student’s *t* test or the Mann–Whitney U test, depending on variable distribution. As a superiority trial, differences in autonomic cardiovascular control and metaboreflex between groups will be tested with two-sided repeated-measures ANOVA. Results will be analyzed as per intention-to-treat. Participants who drop out and do not complete outcome assessment will be compared with those who completed the protocol but their data will be excluded from analysis. Statistical significance will be considered for *P* < 0.05. All analyses will be performed using PASW Statistics 18® (International Business Machines Corp., New York, NY).

### Results communication

A summary of the main findings in non-technical language will be sent to participants after study completion. Scientific papers written according to the CONSORT recommendations will be submitted for peer-reviewed publication. Study results will also be communicated as abstracts and presentations in local, national, and international scientific meetings.

## Discussion

The effect of inspiratory muscle training on blood pressure in hypertensive individuals is not yet established. A significant reduction in blood pressure was observed in a small randomized clinical trial with six and five subject allocated to intervention and control groups, respectively [13]. Although these findings do not provide enough evidence to support the efficacy of inspiratory muscle training in blood pressure control, they are encouraging. We propose an adequately sized randomized clinical trial to test the hypothesis that inspiratory muscle training reduces blood pressure in persons with hypertension. If the hypothesis is confirmed, this study will also allow for investigation of putative mechanisms mediating such an effect. We will assess the effects of inspiratory muscle training on autonomic cardiovascular control and metaboreflex in subjects with hypertension – data that, to the best of our knowledge, are lacking in the medical literature.

The evaluation of inspiratory muscle metaboreflex is one of the strengths of this study. Inspiratory muscle training attenuated the metaboreflex in healthy subjects [30] and in persons with chronic heart failure [31]. In healthy subjects, decreased inspiratory work through assisted ventilation increases exercise time on a cycle ergometer by 14 % [32]; a similar intervention has been shown to attenuate quadriceps fatigue during exercise [9]. This effect is probably due to inhibition of inspiratory muscle metaboreflex [10].

The effects of inspiratory muscle training deserve to be better understood given the potential to help control blood pressure in hypertensive patients. The results of this study will provide evidence on the effects of inspiratory muscle training on blood pressure, in addition to inspiratory muscle metaboreflex, autonomic cardiovascular control, and other physiologic and metabolic aspects. These data will provide mechanistic insights, as well as useful clinical information for the management of hypertension.

## Trial status

The trial is enrolling participants.

## Abbreviations

ANOVA, analysis of variance; CONSORT, Consolidated Standards of Reporting Trials

## Additional file

Additional file 1: Supplementary material. Methods for assessment of study outcomes and other variables. **Figure S1.** Schedule of enrollment, intervention, and assessments. **Figure S2.** Inspiratory muscle metaboreflex induction protocol flowchart. (DOCX 362 kb)
