# Supplementary material for: Effect of inspiratory muscle training with load compared with sham training on blood pressure in individuals with hypertension: study protocol of a double-blind randomized clinical trial
Source: Trials. 2016 Aug 2;17:382. doi: 10.1186/s13063-016-1514-y (PMC4969737; doi:10.1186/s13063-016-1514-y)

*“****Additional File 1 - Supplementary material***

***Methods for assessment of study outcomes and other variables***

- *Ambulatory blood pressure monitoring (ABPM)*

*ABPM is performed with Spacelabs 90207 devices (Redmond, WA, USA). Cuff size (regular or large) is chosen based on brachial circumference. The protocol includes blood pressure (BP) assessments every 15 minutes during daytime (6AM to 10PM) and every 20 minutes during nighttime (10PM to 6AM). ABPM is considered satisfactory if at least 16 valid readings during daytime and 8 valid readings during nighttime are obtained (1). The timeline of outcomes assessment and intervention are depicted in Figure 1S.*

- *Functional capacity*

*A cardiopulmonary exercise protocol tests cardiopulmonary conditioning. It is performed on a treadmill (T-2100 Treadmill, GE Healthcare, Idaho, USA), with a breath-by-breath gas analyzer system (Metalyzer 3B, CPX System, Cortex, Leipzig, Germany), and an aneroid sphygmomanometer. Equipment is calibrated before each test, performed in a room with controlled temperature (19 to 21^o^C) and humidity (relative humidity 60-70%). Participants maintain their usual medical treatment, if any, and receive thorough information about test procedures before the session begins.*

*Test lasts for 8 to 12 minutes in an incremental ramp protocol; this protocol is indicated for individuals with comorbidities or with functional or age-related limitations. Speed is increased by 0.3-0.7 km/min while inclination is increased by 0.5 to 1%/min, depending on the individual’s estimated physical conditioning. Test will be stopped per participant’s request or per criteria described in the American Thoracic Society/American College of Chest Physicians consensus for cardiopulmonary exercise testing (2). Gas fraction analysis in open circuit evaluates peak oxygen consumption (VO_2_, in ml/kg/min) in each breath. Ventilatory threshold is defined as established in the literature (2, 3). Functional capacity is reassessed with the same protocol after the 8 weeks of intervention. In case of intercurrences or emergencies, the test center is equipped with emergency kit including ACLS defibrillator and medications.*

- *Maximum inspiratory pressure (MIP) and maximum expiratory pressure (MEP): respiratory muscle strength*

*Subjects are seated with an angle of 90^o^ between trunk and legs and with a nasal clip in place throughout the assessment. The manometer is connected to a mouthpiece, and participants are instructed to seal the lips around it. The mouthpiece is designed to avoid oral cavity pressure increase generated by undesired contraction of oropharyngeal muscles (4). Participants are instructed to perform diaphragmatic respiration. MIP is then measured during forced inspiration starting from the residual volume; MEP is measured during forced expiration from the total lung capacity. Participants will perform up to 12 maneuvers to provide 6 measurements with variation <10% and avoid effects of learning curve. The highest 1-second peak pressure among the 6 measurements will be used in the analysis (5). MIP and MEP are considered surrogates of inspiratory muscle strength; they will be measured as described above before and after the intervention.*

- *Inspiratory muscle endurance*

*Participants ventilate through a circuit made up by two unidirectional valves with linear pressure resistance (Powerbreathe Plus®, London, UK). An initial load of 50% of MIP is applied. Every 3 minutes the load is increased by 10% of MIP. Test is stopped when subject cannot open the inspiratory valve or desires to stop the test due to respiratory exhaustion, quantified with the modified Borg scale (ranging between 0 – no respiratory fatigue – to 10 – maximal respiratory exhaustion) (6).*

- *Autonomic control*

*BP variability, HR variability and the cardiac vagal tone are assessed to evaluate autonomic cardiovascular control. Non-invasive continuous blood pressure monitoring is used to register pressure waves are detected by a cuff placed on the intermediate phalange of the third finger. After 20 of rest, continuous BP curves, heart rate, and electrocardiogram tracing are obtained with Biopac MP150 (Biopac, California, EUA) at a frequency of 1000Hz and registered simultaneously in a computer with biologic signals conversion capabilities. These signals are subjected to analytic protocols to provide BP and HR variability with the use of fast Fourier transform during exercise phase and in post-exercise rest phase.*

- *Inspiratory muscle metaboreflex*

*The metaboreflex induction protocol consists of causing respiratory muscle fatigue through exercises and measuring leg arterial blood flow reduction simultaneously. Participants are instructed not to perform physical activities for 48h before the test; they should not have caffeinated or alcoholic beverages for at least 12 hours before the test, and they are instructed to fast for 2h before the protocol begins.*

*First, maximum inspiratory pressure is determined for each individual as described above. Subject is then positioned on a bed with elevated head (semi-supine), and monitoring of physiologic parameters begins. Respiratory rate and peripheral O_2_ saturation is monitored with pulse oximetry. Heart rete is monitored with electrocardiography tracing. An automated sphygmomanometer cuff (Dinamap, DASH 2000, General Electric, CT) is placed on the non-dominant arm over the brachial artery to measure systolic, diastolic, and mean arterial blood pressures. Blood pressure and heart rate variability are assessed as described above (‘autonomic control’ section). End-tidal CO_2_ partial pressure is monitored with infrared capnography (Takaoka, USA).*

*Blood flow is determined by venous occlusion plethysmography (Hokanson, WA) in the non-dominant lower limb every 10 seconds, expressed in ml/min/100ml. Simultaneously, mean arterial blood pressure will be monitored with automated sphygmomanometer (Dinamap, DASH 2000, General Electric, Bloomfield, CT, USA) on the popliteal artery contralateral to the limb where blood flow is measured. Vascular resistance is calculated dividing mean arterial blood pressure by blood flow.*

*After 15 minutes of rest, baseline autonomic control assessment begins. A nasal clip is placed and participant is instructed to breathe through a two-way respiratory system (Hans Rudolph, KS). Phase 1 of this assessment consists of 10 minutes of spontaneous ventilation; phase 2 consists of 10 minutes of controlled respiratory rate (15rpm). After this step, subjects have a 30-minute break.*

*After the break, metaboreflex induction protocol begins. Subjects are instructed to keep spontaneous breathing for 5 minutes, while baseline measurements are recorded. A breathing trainer (PowerBreath, UK) is then connected to the respiratory system to create an inspiratory load equal to 60% of the maximum inspiratory pressured assessed at the beginning of the session. After that, a period of controlled ventilation starts.*

*Participants should keep respiratory rate at 15rpm and the ratio inspiratory time/total cycle time at 0.75. A metrometer designed by the Biomedical Engineering Service at Hospital de Clínicas de Porto Alegre aids subjects to maintain the correct rhythm through sounds and lights that signal for inspiration and expiration. During each cycle, subjects are instructed to 1) perform diaphragmatic breathing to avoid use of accessory muscles, and 2) maintain the inspiratory pressure constant, with the aid of visual feedback as the inspiratory pressure is continuously recorded and displayed on a screen. Modified Borg scale assesses respiratory effort every 1 minute during the exercise. Borg’s scale quantifies perceived exertion at the end of the protocol.*

*Metaboreflex induction exercise with 60% MIP load ends when: 1) subject is unable to open the resistance device’s valve; or 2) a pressure less than 90% of the predicted MIP is observed in 3 consecutive breaths. When either of the criteria is met, respiratory device is unloaded and participant is instructed to keep the same respiratory pattern guided by the metrometer. Subject is then monitored for 10 minutes in this recovery phase.*

*After a 40-minute break, the sham protocol starts with 2% MIP load. The same procedures adopted for the induction protocol are repeated. The duration of the sham protocol is determined by the duration of the induction protocol for each participant.*

***Figure S1:*** *Schedule of enrollment, intervention, and assessments.*

***Figure S2:*** *Inspiratory muscle metaboreflex induction protocol flow-chart”*

(please see bellow)

Figure S1: Schedule of enrollment, intervention, and assessments

|  | **STUDY PERIOD** | | | | | |
| --- | --- | --- | --- | --- | --- | --- |
|  | Enrolment | Allocation | Baseline assessment | Intervention period | Primary endpoint | Close-out |
| **TIME POINT** | Week -2-0 | | Week 0 | Week 1-8 | Week 8 | Week  9-12 |
| **ENROLMENT:** |  |  |  |  |  |  |
| Eligibility screen | **X** |  |  |  |  |  |
| Informed consent | **X** |  |  |  |  |  |
| Allocation |  | **X** |  |  |  |  |
| **INTERVENTION:** |  |  |  |  |  |  |
| Inspiratory muscle training |  |  |  |  |  |  |
| Sham respiratory training (control group) |  |  |  |  |  |  |
| **ASSESSMENTS:** |  |  |  |  |  |  |
| IPAQ, RMS, height, weight |  |  | **X** |  |  |  |
| ABPM |  |  | **X** |  | **X** |  |
| Metaboreflex inspiratory |  |  | **X** |  |  | **X** |
| Functional capacity |  |  | **X** |  |  | **X** |
| Respiratory muscle strength tests |  |  | **X** | **X** | **X** | **X** |
| Respiratory muscle endurance |  |  | **X** |  |  | **X** |

IPAQ: International Physical Activity Questionnaire; RMS: respiratory muscle strength; ABPM: ambulatory blood pressure monitoring.

**Figure S2:** Inspiratory muscle metaboreflex induction protocol flow-chart


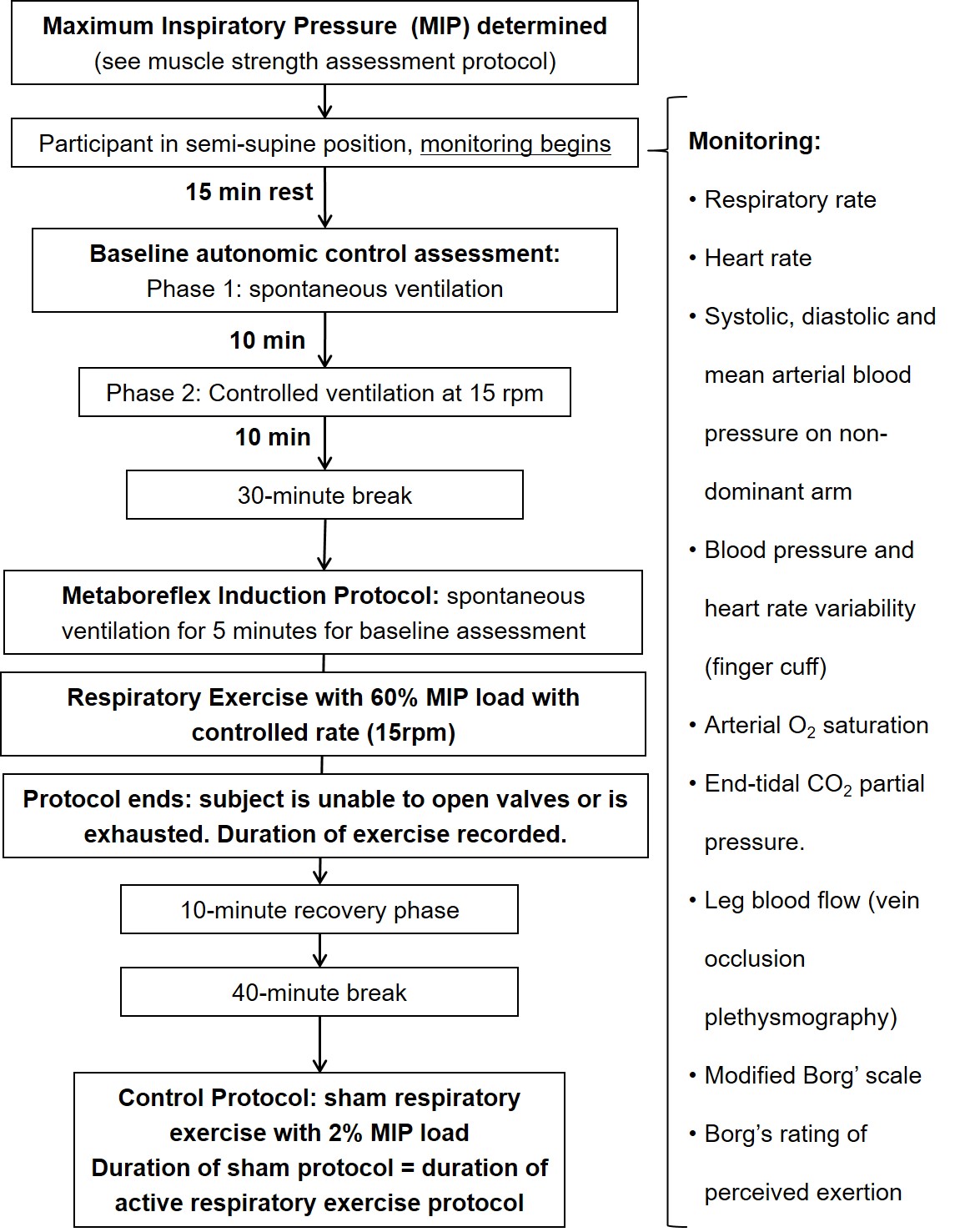

Supplement: Supplementary file 1 — Supplementary material. Methods for assessment of study outcomes and other variables. Figure S1. Schedule of enrollment, intervention, and assessments. Figure S2. Inspiratory muscle metaboreflex induction protocol flowchart. (DOCX 362 kb) [file 13063_2016_1514_MOESM1_ESM.docx]
